# HIV Latency in Myeloid Cells: Challenges for a Cure

**DOI:** 10.3390/pathogens11060611

**Published:** 2022-05-24

**Authors:** Alisha Chitrakar, Marta Sanz, Sanjay B. Maggirwar, Natalia Soriano-Sarabia

**Affiliations:** Department of Microbiology, Immunology and Tropical Medicine, The George Washington University, Washington, DC 20037, USA; alishachitrakar@gwu.edu (A.C.); martasanzp@gwu.edu (M.S.); smaggirwar@gwu.edu (S.B.M.)

**Keywords:** HIV latency, HIV cure, cellular reservoirs, monocytes, macrophages, CNS, myeloid cells

## Abstract

The use of antiretroviral therapy (ART) for Human Immunodeficiency Virus (HIV) treatment has been highly successful in controlling plasma viremia to undetectable levels. However, a complete cure for HIV is hindered by the presence of replication-competent HIV, integrated in the host genome, that can persist long term in a resting state called viral latency. Resting memory CD4+ T cells are considered the biggest reservoir of persistent HIV infection and are often studied exclusively as the main target for an HIV cure. However, other cell types, such as circulating monocytes and tissue-resident macrophages, can harbor integrated, replication-competent HIV. To develop a cure for HIV, focus is needed not only on the T cell compartment, but also on these myeloid reservoirs of persistent HIV infection. In this review, we summarize their importance when designing HIV cure strategies and challenges associated to their identification and specific targeting by the “shock and kill” approach.

## 1. Introduction

### Definition of Viral Reservoir

Prevention of HIV replication by simultaneously targeting several steps in the viral cycle with antiretroviral therapy (ART) allows to reduce plasma viremia below the limit of detection in clinical assays. However, ART does not eliminate HIV, and after depletion of CD4+ T cells during primary infection, some cells enter a resting state where HIV can remain persistently integrated into the host cell genome [1,2,3]. This resting state, where HIV is not actively replicating, is called viral latency. Therefore, viral reservoirs are defined as cells that harbor integrated replication-competent HIV that persist in long-lived cells during suppressive ART. Multiple mechanisms are involved in establishing such latency and have been discussed elsewhere [4]. Resting memory CD4+ T cells are the most widely studied cellular reservoir of persistent HIV and they are extremely stable over time [5,6]. HIV persists in all subsets of CD4+ T cells including memory and naïve cell subpopulations although distinct transcriptional activity and integration sites can contribute to variable viral inducibility following reactivation [7,8,9,10,11]. Contributors to HIV persistence within CD4+ T cells during ART include homeostatic or antigen-driven proliferation and possibly ongoing viral replication that maintains the immune system activated and exhausted [7,12,13,14,15,16,17,18,19]. These elements constitute the main barrier to cure.

In addition to CD4+ T cells, other cell types might serve as HIV reservoirs, including γδ T cells that constitutively express CCR5 [20]. Despite generally lacking CD4 expression, γδ T cells transiently upregulate CD4 expression upon activation, become targets for HIV infection and can serve as reservoirs harboring replication-competent HIV [21,22]. In addition, other non-T cell subpopulations, including monocytes and macrophages, are susceptible to HIV infection and allow long-term persistence of HIV in people living with HIV (PLWH) on optimal ART. This article reviews main characteristics of human circulating and tissue-resident myeloid cells, their role as reservoirs and potential issues for current strategies to cure HIV infection. Finally, we discuss challenges and strategies that may help overcome killing resistance by effector cytotoxic cells as part of the shock and kill approach.

## 2. Myeloid Cells: Origin, General Characteristics and Subpopulations

**Figure 1:**
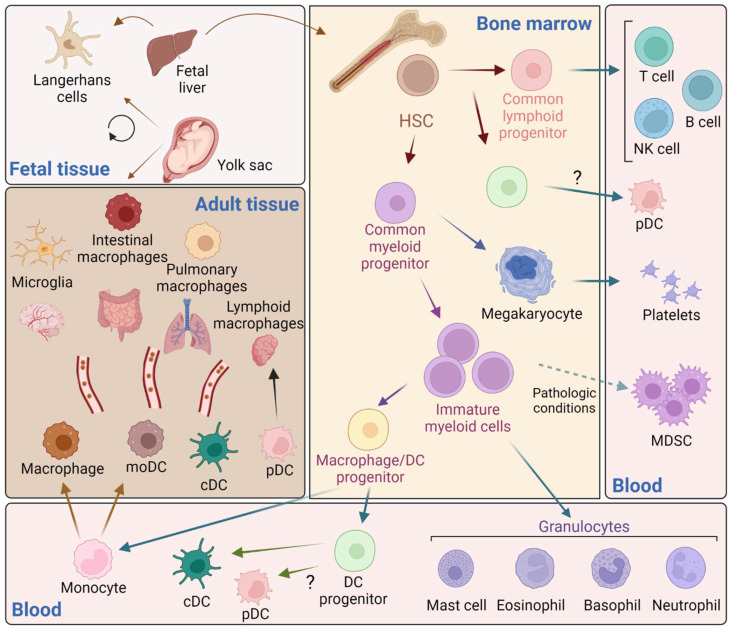
Schematic representation of white blood cells’ ontogeny. Tissue-resident macrophages with self-renewal capacity originate from the yolk sac (i.e., pulmonary, intestinal, brain (microglia), lymphoid, skin (Langerhans) during embryogenesis and populate their respective tissues. Langerhans cells also derive from the fetal liver, which produces hematopoietic stem cells (HCS) that colonize the bone marrow. HSC give rise to both common lymphoid and myeloid progenitors with capacity to produce megakaryocytes, precursors of platelets. Common myeloid progenitor cells also give rise to a heterogeneous population of immature myeloid cells that rapidly differentiate into granulocytes and a macrophage/dendritic cell (DC) precursor. Monocytes migrate to replenish the tissues and mature into macrophages and monocyte-derived DC (moDC) that as part of the normal homeostasis and surveillance. DC progenitors give rise to conventional DC (cDC) and plasmacytoid DC (pDC), although it is not clear whether pDC arise from an intermediate precursor prior to the generation of common lymphoid progenitors (recently reviewed in reference x). Under pathological conditions (e.g., infection, tumor, inflammation) immature myeloid cells produce myeloid-derived suppressor cells (MDSC) (Figure created with Biorender).

Hematopoietic stem cells in the bone marrow produce two different progenitor cells that will give raise to the lymphoid and myeloid lineages (Figure 1). Immature myeloid cells are intermediate precursors of the normal process of myelopoiesis that comprise a heterogeneous population of common myeloid progenitor cells that rapidly differentiate into mature myeloid cells. However, in several pathological conditions, immature myeloid cells with suppressive activity, known as myeloid-derived suppressor cells, are expanded and mediate suppression of T cell functions [23,24].

Mature myeloid cells belong to the innate immune system and comprise several subpopulations classified into mononuclear and polymorphonuclear cells (or granulocytes). Mononuclear phagocytes include monocytes, macrophages, and dendritic cells (DCs), and granulocytes include neutrophils, eosinophils, mast cells and basophils [25]. Granulocytes, and neutrophils specifically, are the most abundant leukocytes with critical antimicrobial functions. As the first responders to infection, they are rapidly recruited to sites of infection and they are short-lived with a circulating half-life of 6–8 h [26]. Monocytes constitute the circulating precursors of macrophages, which reside virtually in all tissues. Monocytes play a major role in protective immunity by circulating through the blood and the lymphatic system, where they are recruited to sites of tissue injury, activated to secrete inflammatory cytokines and to phagocyte [27]. Tissue-resident macrophages perform immune surveillance, homeostatic and tissue repair functions [28,29]. Monocytes had been long considered a developmental intermediate between precursors of the bone marrow and mature tissue macrophages. However, it has become clear now that both circulating monocytes recruited to tissues contribute to in situ homeostasis [30,31], and many tissue-resident macrophage populations locate in those tissues and are homeostatically maintained [31,32,33,34,35]. Early observations have shown that macrophages exist in the embryonic yolk sac prior to the formation of hematopoietic stem cells and therefore are seeded within tissues during embryogenesis [36]. However, that origin has been neglected for decades and the idea that blood-derived monocytes were the only cells responsible for tissue-macrophage replenishment prevailed leading to misconceptions on monocyte-macrophage origins and functions. Currently, the embryonic origin of macrophages is widely demonstrated and accepted [33,37,38,39]. In addition, embryonic cells with myeloid cell potential can migrate to the fetal liver and differentiate into progenitor cells and are considered the key progenitor cells for developing tissue-specific macrophages [36,37].

This improved understanding of the myeloid population immunobiology and function has been fundamental to expand investigations towards characterizing how the HIV reservoir is established, maintained and reactivated in specific monocytes and macrophage populations. The relevance of such a reservoir remains poorly characterized and understood despite being a critical component for HIV cure strategies due to their longevity and self-renewal properties.

## 3. Circulating Monocytes and Tissue-Resident Macrophages Subpopulations and Characteristics

Circulating monocytes can be identified by flow cytometry using CD14 and CD16 monoclonal antibodies. They are primarily classified into three phenotypically and functionally distinct subpopulations: classical (90%, CD14^++^CD16^−^), intermediate (5%, CD14^++^CD16^+^) and non-classical (5%, CD14^+^CD16^++^) monocytes [40,41,42]. Further work has defined additional monocyte subpopulations with specific epigenetic and cytokine production signatures, providing an extra layer of complexity within the monocyte lineage [43,44]. In addition, age and sex may impact the interindividual distribution of monocyte/macrophage subpopulations with specific phenotype and functions [43,44,45,46], and was recently reviewed [47,48]. Immune cells’ trafficking to be recruited to sites of injury, infection or transformation is governed by the level of chemokine receptors’ expression on a given cell population, and the balance of chemokine and cytokine levels present in the environment [49,50,51,52]. As such, phenotypic signatures of the three primary monocyte subpopulations govern their migration capabilities based on expression levels of chemokine receptors.

Classical monocytes express high levels of CCR2 and low levels of CX3CR1 and migrate in response to CCL2 chemokine gradients, while CD16^+^ monocytes express low CCR2 and high CXCR1 levels and migrate towards CXCL1 (fractalkine) gradients [53,54]. Intermediate monocytes have lower expression of CCR2 and CXCR1, and higher expression of CX3CR1 compared to classical monocytes. MHC-II expression is higher in intermediate monocytes than both the classical and non-classical population. Expression patterns of these chemokine receptors among the three main monocyte subpopulations are linked to specific functions. Classical monocytes migrate to sites of injury and differentiate into inflammatory macrophages, whereas non-classical monocytes exhibit immune surveillance functions along the blood vessels. Both non-classical and intermediate monocytes are the source of proinflammatory cytokines, such as TNF-α, IL-1β, IL-6 and IL-18, with intermediate monocytes producing significantly more TNF-α and IL-1β than the other populations.

Tissue-resident macrophages have critical roles as first line of defense against intruding pathogens, maintaining homeostasis, and tissue integrity. Their phagocytic function is crucial for both uptake and clearance of injured, dying or cellular debris, and pathogenic microbes [28,55]. Located in different anatomical areas and organs, macrophages require tight regulation by transcriptional mechanisms, regulated both by origin and tissue microenvironment [56]. Some tissue-resident macrophage populations, including microglia and Langerhans’ macrophages, have self-renewal capacities and therefore can be maintained independently of monocytes, although circulating monocytes migrate to tissues and can acquire the phenotype of resident macrophages [28,34,57,58,59,60]. The extent of the contribution of tissue-resident vs. circulating monocytes to the maintenance of local macrophage populations is currently unknown since trauma, injury or chronic inflammation can affect monocyte infiltration. It seems clear though that the half-lives of certain macrophage populations can be similar to memory T cells and therefore their contribution to the persistence of HIV during suppressive ART and their critical importance for HIV cure strategies must be addressed.

## 4. HIV Infection and Reservoirs in Monocytes/Macrophages

Although CD4+ T cells are the main target of HIV infection, macrophages express low levels of CD4 and their susceptibility to productive infection was described more than 2 decades ago [61]. However, due to the diversity of macrophage subpopulations, relatively low virus production and insufficient understanding of their development, immunobiology and diversity has dampened our general understanding of the role of monocytes/macrophages in HIV pathogenesis [48,62]. Macrophages likely play two opposing roles in the acute phase of HIV infection. On the one hand, they help to establish infection at sites of viral entry, and monocytes and perivascular macrophages disseminate the virus throughout the body, including the brain [63]. The description of tissue-resident macrophages, wider understanding of their self-renewal properties and increased resistance to cytopathic effects compared to CD4+ T cells, has renewed the interest in the field to investigate their role as latent HIV reservoirs. Since permissiveness to HIV infection differs among macrophage populations, depending on the localization and sample size, the identification of latent macrophage reservoirs might be challenging [64,65,66,67].

## 5. HIV Latency in Monocytes

Circulating monocytes in the blood have a shorter lifespan (~4 to 7 days) and they differentiate into macrophage phenotype soon after their extravasation into the tissues [68]. Their role in maintaining a long-term persistent HIV reservoir has therefore been controversial [69]. However, it is well established that monocytes are infected in the acute stages of HIV infection and they play a role in establishing HIV-associated neurocognitive disorder (HAND) [70]. Classical monocytes, expressing low levels of CD4 and CCR5 receptors, are less susceptible to HIV infection than intermediate or non-classical monocytes. Both monocyte subpopulations expressing CD16, have higher levels of CCR5 expression, which makes them more susceptible to HIV infection [71,72]. Therefore, identification of the HIV reservoir within monocytes requires a deeper phenotypic characterization.

Early and late, but not integrated, HIV reverse transcripts were detected in blood monocytes of a small population of ART- suppressed PLWH, whereas HIV DNA was only found in CD4+ T cells [69]. Monocytes have host-restriction mechanisms that limit HIV infection, including SAMHD1 and APOBEC3 [73]. These restriction factors are proteins that provide an initial line of defense against HIV infection [74,75]. Despite the presence of these restriction factors in myeloid cells, integrated HIV DNA and replication-competent virus were detected in some studies performed in PLWH on suppressive ART [76,77,78].

A recent study investigating cellular reservoirs of HIV-1 in donors undergoing analytic treatment interruption (ATI) also identified macrophage-tropic HIV-1 variants in the plasma of ART-suppressed PLWH. Molecular clock analysis also suggested that these variants were potentially established before ART interruption [79]. These studies present us with further evidence that macrophages need to be considered as critical cellular reservoirs in HIV cure strategies.

## 6. HIV Latency in Tissue-Resident Macrophages

Even though the role of circulating monocytes as a reservoir of HIV is debatable, tissue-resident macrophages have long known to be cellular reservoirs because of their presence in multiple tissues and their long lifespan.

Gut macrophages were previously believed to be resistant to HIV infection and were thought to play a role in generating antibody responses against the virus to decrease plasma viremia in the initial stages of infection [80]. However, using gut biopsy samples from ART-suppressed PLWH, studies have shown that duodenal macrophages express p24 and harbor proviral DNA [38,81]. Similarly, urethral macrophages in ART-suppressed PLWH were also shown to be the primary cells to harbor HIV-1 reservoir in penile urethra [82]. In these macrophages, containing total and integrated HIV-1 DNA, LPS stimulation reversed latency leading to expression of HIV-1 p24 protein [82,83].

HIV-1 DNA was also detected in specialized macrophage Kupffer cells from the liver of PLWH postmortem, although studies in SIV-infected macaques suggest that the liver is not the primary site of viral replication [84,85]. Using bronchoalveolar lavage (BAL) of ART-suppressed PLWH, HIV RNA and proviral DNA was detected in alveolar macrophages in some but not other patients [86,87]. The presence of proviral DNA in alveolar macrophages displayed impaired phagocytic capacity compared to healthy controls, which is consistent with the observations in ART naïve PLWH [86,88]. This suggests the latent alveolar macrophages are impairing pulmonary immunity, making PLWH more susceptible to respiratory tract infections [86,89].

There have been contradicting reports on the role of Langerhans cells during HIV infection. Numerous reports have shown that Langerhans cells can uptake HIV and were previously hypothesized to mediate viral transmission to CD4+ T cells [82,90,91,92]. However, recent studies have contradicted these reports by showing Langerhans cells prevented HIV infection via actions of C-type lectin Langerin, thus preventing further transmission [93]. Regarding the role of Langerhans as additional HIV reservoirs, studies concluded that they may not be a principal reservoir, although some evidence suggests that this may be HIV-subtype dependent [94,95]. Vaginal epithelial dendritic cells (CD1a^+^ VEDCs) were recently defined to be a separate subset that were previously misclassified as Langerhans cells. CD1a^+^ VEDCs isolated from virologically suppressed women harbored HIV-1 DNA, suggesting their potential as an HIV reservoir [96]. The most significant monocytic reservoirs of HIV were noted in the CNS, which will be discussed in detail below.

Collectively, there is significant evidence for the role of macrophages as reservoirs in ART-suppressed individuals that warrants further studies to characterize them in ATI interventions aimed at HIV eradication.

## 7. Models to Studying Reactivation of Myeloid HIV Reservoirs

Investigation of HIV latency in human tissue-resident macrophages is challenging due to their anatomical location that is limited to biopsies post-surgery or postmortem samples. Despite the inherent differences with primary cells, in vitro studies from latently infected monocytic cell lines and animal models are useful as a proxy to better understand mechanisms of viral latency and reactivation.

### 7.1. Latently Infected Monocytic Cell Lines and Primary Cells

Infection of some myeloid cell lines leads to survival of those carrying integrated proviruses [97]. Here, we briefly review some of the latently infected cell lines and reports on susceptibility to reactivation.

U1 promonocytic cells, developed by Fauci’s group, are derived from the U937 parent cells infected with replication-competent HIV-1 (LAV-1 strain) that contain two copies of integrated proviral DNA per cell. These U1 cells have minimal constitutive expression of HIV but virus can be produced upon stimulation with phytohemagglutinin (PMA) and proinflammatory cytokines such as interleukin (IL) IL-1α, IL-1β, IL-6 and TNFα [98,99,100].

The monocytic cell line (THP-1) was derived by culturing the blood of a patient with acute monocytic leukemia by Tada’s group [101]. Upon stimulation with PMA, THP-1 cells have a monocyte-derived macrophage (MDM)-like phenotype with an activation of CD16 and IL-1β. Constitutive expression of CD4, CCR5 and CXCR4 makes them susceptible to HIV-1 infection [97,102,103]. This cell line has been used to describe viral latency and to test reactivation of the virus using LPS, PMA, TNFα and GM-CSF. Transcriptional silencing can be reverted inhibiting DNA methylation by 3-deaza-adenosine [97,104,105].

HC69 are latently HIV-infected human microglial cell lines developed by Karn’s group. They were developed by immortalizing human microglial cells (C20 cell line) using simian virus 40 large T antigen (SV40Tag)/human telomerase reverse transcriptase (hTERT) [106]. These immortalized C20 cells were then infected with a vesicular stomatitis virus G (VSV-G) envelope pseudotyped lentivirus vector (PHR1′/d2EGFP), thus expressing a green fluorescent protein (d2EGFP) as a reporter [106]. Although these cells exhibit a background level of spontaneous GFP expression in culture, latency of HC69 cells can be effectively reversed by using TNFα and poly (I:C) TLR3 agonist [106,107].

Human primary monocyte models may constitute more relevant in vitro models for evaluation of HIV cure strategies [108]. Monocytes isolated from peripheral blood mononuclear cells (PBMC) can be differentiated in vitro into macrophages (MDM) using macrophage colony stimulating factor (MCSF) or granulocyte macrophage colony stimulating factor (GM-CSF) to induce anti- or pro-inflammatory macrophage phenotypes [108,109,110,111]. MDMs can also use other stimuli to polarize them, such as IFNγ and TNFα for M1 (classical) or IL-4 for M2 (non-classical) phenotype [108,112,113,114,115]. Different polarization stimuli can influence HIV infectivity and reactivation in these models [108,112,113,114,115].

The polarized MDMs can be infected with M-tropic eGFP tagged reporter HIV to generate latency models in vitro [108,116]. Brown et al. described an MDM model where these infected MDMs were cultured for up to 78 days post infection and observed that the FACS purified GFP- MDMs harbored proviral DNA, which could be reactivated by IL-4 and PMA to produce replication-competent HIV [116]. Wong et al. recently developed an improved primary model where they generated latently infected MDM model by culturing the infected MDMs with T20 (enfuvirtide) to prevent de novo infection and FACS-sorted GFP-MDMs nine days after infection did not show HIV-1 p24 production. These sorted GFP-MDMs could, however have a background level of spontaneous reactivation in culture. They also showed that the polarizing stimulus before infection modulates the latency reactivation, with M2 polarized MDMs reactivating at a higher rate than M1 polarized MDMs [108]. The latently infected MDMs were used to study modulation of latency reversing agents and are discussed in a section below [108].

These cell lines and in vitro models of latent infection have been popular due to their ease to culture, short time commitment and cost effectiveness. They have been important in demonstrating effectiveness and mechanisms of various molecules used as ART and LRA. However, they are not sufficient for studying systemic effects of targeted therapies, which would require the use of animal models. Especially for studying tissue-resident macrophage reservoirs of HIV, the use of animal models is irreplaceable.

### 7.2. Humanized Mice

Humanized mice are generated using immunodeficient mice transplanted with human cells, tissues or both, therefore, allowing to be HIV infected while recapitulating some aspects of the human immune system. These models allow experimental interventions and tissue sampling that are not possible in clinical studies. This is particularly true for the bone marrow–liver–thymus (BLT) mouse where both CD4+ T cells and myeloid cells can be investigated in the context of HIV infection, pathogenesis and cure. One critical limitation of studies involving humanized mice models is the small size of the mice requiring, in general, pooling of several animals to achieve enough sensitivity to quantify viral reservoirs. The different types of humanized mouse models used to study HIV persistence and their benefits and limitations have been recently reviewed and are only briefly described here [117,118,119].

Peripheral blood lymphocyte (PBL)-humanized (hu)-mice can be generated by reconstituting various strains of immunocompromised mice, such as NOD/SCID/IL2Rγ-null (NSG), lacking mature T, B or NK cells and transplanting with human PBMCs. These PBL-hu mice show reconstitution of CD4+ and CD8+ lymphocytes within 6–8 weeks of transplant [120]. PBL-hu-mice are an effective model because of the ease and time of generation. They provide a good model for HIV infection and prevention, but since they are susceptible to rapid Graft-versus-Host disease (GvHD), they cannot be used as a long-term infection model [121]. Human macrophages are also not incorporated in this model, which makes them a poor model for studying macrophage HIV reservoirs [120,122].

Various strains of immunocompromised mice can be reconstituted with human CD34+ hematopoietic stem cells (HSC) from multiple sites of origin (fetal liver and thymus tissues and neonatal cord blood) to generate an HSC-hu mouse model. These CD34+ hu mice can be infected with HIV and upon ART initiation, viral load is suppressed [123]. HIV establishes reservoirs in various tissues, and macrophage reservoirs of HIV were detected in the spleen and bone marrow [124,125]. Limitations of this model include poor lymph node and spleen reconstitution in the mice, including limited development of the T cell, B cell and myeloid cells [126,127,128]. Reports of mucosal transmission has been shown to be dependent on the strain of mice and the origin of CD34+ HSC [129,130].

The development of humanized mice by Garcia’s group, called myeloid only mice (MoM) was instrumental in studying the HIV reservoir in macrophages in the absence of CD4+ T cells. These mice were generated in NOD.CB17-Prkdc^scid^/J mice (NOD/SCID) by transplanting with human CD34+ hematopoietic stem cells (HSC). These mice were reconstituted with B cells and myeloid cells and were completely devoid of T cells [131]. Using this model, authors further demonstrated that tissue macrophages are a reservoir of persistent HIV during suppressive ART [132].

The most robust humanized model of HIV is the bone marrow, liver, thymus (BLT) mice. Hu-BLT mice are generated by implanting human thymus and liver tissues under the kidney capsule of immunocompromised mice like NSG or C57BL/6 Rag2^−/−^γc^−/−^CD47^−/−^ (TKO) mice and injecting autologous CD34+ HSC simultaneously [128,133,134]. The main advantage of BLT mice is the presence of a human thymic environment and systemic human cell reconstitution, including the mucosal tissues, making it an excellent model for studying mucosal transmission of HIV [135,136,137,138,139,140]. The hu-BLT model is the most suitable for HIV persistence studies due to their ability to establish and maintain latent reservoirs after extended periods of ART [135,141,142]. In addition, they were also used to demonstrate the establishment of viral reservoirs in the CNS that persist during ART [143]. However, there are limitations of this model since they are susceptible to GvHD starting six months post reconstitution [144]. There are improved BLT mouse model incorporating human spleen called the bone marrow–thymus–liver–spleen (BLTS) mice that showed improved GvHD instance and better reconstitution of T cell, B cell and macrophages [144,145]. Regardless, hu-BLT/BLTS mice still require the use of fetal tissue which makes them harder to generate, in addition to other general limitations of humanized mice, such as their small size, limited lifespan and the variability in immune cell lineage differentiation during reconstitution [118].

Humanized mouse models are excellent in vivo models for initial HIV cure studies and can successfully mimic results observed in non-human primate models, as reported for the use of reverse latency agents [146].

### 7.3. Non-Human Primates

Non-human primates (NHPs) are the only other species that are naturally susceptible to HIV infection. There are different strains of Simian Immunodeficiency Virus (SIV) that causes different disease phenotypes depending on the species of NHPs, which have been reviewed recently [147].

In infectious SIV model, infection of NHP with SIV leads to disease progression and immunopathogenesis that bears close resemblance to HIV infection in humans. That includes CD4+ T cell depletion, viremia that can be controlled using ART and ATI resulting in viral rebound [147,148]. However, the use of cross species SIV is shown to be most effective in infection progressing to AIDS, i.e., infection with one strain of SIV will not cause severe disease in some NHP (natural host) but would cause AIDS in a different NHP (non-natural host) [149]. This reduced pathogenesis in natural host was predicted to be due to the lack of macrophage infection [150].

The level of macrophage infection in most NHP models are detectable but are highly elevated upon CD4+ or CD8+ T cell depletion [151]. Infection of macrophages is greatest in pathogenic SIV conditions in non-natural hosts [150]. Various macrophage-tropic SIV strains are discussed in the review by Moeser et al. [152]. Briefly, SIVmac239, SIVsm804E-CL757, SIV/17E-Fr and SIVsmE543-3 were shown to efficiently infect macaque macrophage in vivo, and SIVmac316 could not infect macaque macrophages. However, there are conflicting reports regarding SIVmac251 infecting macrophages without depletion of CD4+ or CD8+ T cells [151,152,153,154,155,156,157,158,159].

SIV-macaque models have been used to detect monocyte/macrophage reservoirs in ART suppressed hosts by using modified quantitative viral outgrowth assay (qVOA) [155,160,161]. These models were also used to study the establishment of myeloid HIV infection. There are reports showing macrophage reservoirs established due to phagocytosis of infected CD4+ T cells, as well as defining the role of circulating monocytes establishing SIV infection in the brain and neurodegenerative disorders [162]. Infecting macaques with neurotropic- and macrophage-tropic SIV strains show disease pathology of SIV encephalitis (SIVE), which is similar to HIV-associated neurocognitive disorders (HAND) in humans [163]. There are also various ongoing studies in various NHP models of HIV to study the establishment of myeloid/macrophage infection. Due to these similarities, the SIV-macaque model has been a valuable tool for developing ART, testing LRA, vaccine development and developing therapeutics in combating comorbidities associated with PLWH the quest to cure HIV.

## 8. The Challenge of HIV Reservoirs in the Central Nervous System

Similar to the notion discussed above, specialized macrophages that reside in the CNS constitute a heterogeneous mixed population of microglia, perivascular, meningeal and choroid plexus macrophages, in addition to monocytes with capacity to infiltrate into the brain under specific conditions [164]. For decades, the concept that microglia originated from circulating monocytes dominated despite initial consideration that they developed during embryogenesis [31,165]. It has recently become clear that microglia, similar to other tissue-resident macrophages, do not arise from blood monocytes but they are distinct functional populations [34,58,166,167]. These long-lived cells with self-renewal capacity and increased resistance to cytopathic effects constitute a formidable potential reservoir of persistent HIV infection. In addition, the long-believed “static status” of microglial cells has been demystified and its active role in homeostatic functions and tissue maintenance and integrity has been recently demonstrated [164,168]. Finally, the immune-privilege status of the brain was challenged after the discovery of a functional lymphatic system in the CNS that drains into peripheral lymph nodes [169,170].

All these advances in our knowledge of the brain-macrophage resident cells are just starting to influence our concept of how HIV infects, establishes productive infection, and maintains cellular reservoirs of HIV in the CNS. The recovery of HIV DNA from brain tissues isolated from autopsies of people living with HIV (PLWH) on suppressed ART, confirmed the presence of CNS reservoirs [171,172]. The question is then, how does HIV establish and maintain such reservoirs?

## 9. HIV Infection and Maintenance of Reservoirs in the Brain

We do not totally understand how HIV disseminates in the brain, although available literature suggests that multiple non-exclusive mechanisms may be involved [173]. However, recent clinical evidence suggests that HIV invasion of the CNS occurs as early as 8 days after estimated exposure, with similar observations reported in SIV-infected macaques, which appears to be associated with increased cellular infiltration [158,174,175,176]. Lack of an ideal model to investigate reservoirs in the CNS and strategies to target and reduce them have been hampered by the lack of physiologically relevant models to resemble the complex human brain and the associated pathologies.

HIV productive infection has been shown in microglial cells, perivascular macrophages and choroid plexus from PLWH [177]. Myeloid cells express low levels of the CD4 receptor and therefore are susceptible to infection. During early infection, main HIV strains are macrophage (M)-tropic with tropism for the CCR5 receptor, that is constitutively expressed in myeloid cells and can also be upregulated during monocyte differentiation [178]. A deeper characterization of brain resident macrophages could provide further insights into specific populations with enhanced susceptibility to HIV infection, similar to recent studies that found a population of gut-resident macrophages with increased CD4 expression in the intestine [179]. In addition, monocyte-derived macrophages that replenish the brain as a normal homeostatic process display increased susceptibility to HIV infection compared to circulating monocytes serving as the Trojan horse model of neuroinvasion [173,180]. Finally, multiple studies have shown that neurotropic viruses adapt and evolve increased capacity to infect macrophages and in fact, genetic compartmentalization is found within the virus from the cerebrospinal fluid (CSF) [177].

Our lab is working on a novel concept of viral dissemination involving platelets. In vitro studies demonstrated that platelets could interact with HIV by harboring HIV particles within their canalicular system or processed virions in endosomal compartments [181,182]. Studies in chronically infected PLWH showed that platelets could bind infectious HIV and infect macrophages [183,184]. More recently, we and others confirmed a potential critical role of platelets in HIV infectivity and latency. Real et al. confirmed that platelets from ART-suppressed PLWH harbor replication-competent HIV [185], and Simpson et al. showed that platelets can promote infectivity by forming complexes with monocytes and CD4+ T cells [186]. Platelet infection could result from active thrombopoiesis of HIV-infected megakaryocytes, as recently suggested or via spontaneous uptake of HIV virions by platelets in the circulation of viremic patients as subsets of platelets express DC-SIGN and CLEC-2 [181,185,187,188,189,190]. It is possible that both events contribute to HIV-positive platelets in viremic individuals, resulting in higher levels of platelet-bound virions pre-cART, and only a decrease, not eradication, following treatment. Nonetheless, these studies set up an interesting novel notion of viral dissemination to the CNS by HIV-infected platelet–monocyte complexes infiltration in the brain that is currently being investigated.

The introduction of highly active antiretroviral therapy (HAART) led to a remarkable reduction of HIV associated dementia (HAND) and control of HIV replication in the CNS [191,192]. However, in the post-HAART period, there has been a rise in milder forms of dementia possibly associated to toxicity related to the continuous use of ART [193,194,195,196,197]. Even in the context of plasma viral suppression, some PLWH experience CSF/plasma discordance, meaning that HIV RNA levels are higher in CSF than in plasma, raising concerns about drug penetrance in the brain and CSF viral escape [176,198,199,200,201,202]. Therefore, maintenance of viral reservoirs in the CNS may be through several mechanisms, including low-level ongoing viral replication or controlled migration of circulating infected cells to the brain as part of the normal immune surveillance [203,204,205,206]. In this regard, the HIV-infected CD14^+^CD16^+^ monocyte subpopulation preferentially migrates towards the CNS and contributes to viral persistence in the brain [207].

## 10. Reactivating Latency from Macrophage Reservoirs

ART maintains undetectable viremia in PLWH, although eradication of the virus is not possible due to the presence of latent reservoirs including cells from the monocyte/macrophage lineage. We have discussed above how macrophages are an important HIV reservoir as they have the capacity to harbor viruses and produce infectious virions. One of the most studied strategies for the cure of HIV is the so-called “shock and kill”, although mostly focus on targeting CD4+ T cell reservoirs [208]. For this purpose, latency reversal agents (LRAs) are used to reactivate latently infected cells, it had been proven that LRAs can efficiently reactivate latent CD4+ T cells [208]. However, evaluation of LRA function on macrophage populations is critical since the transcription factors that regulate HIV latency and maintenance in macrophages are distinct from those in CD4+ T cells [209,210]. In addition, inherent enhanced resistance to apoptosis and HIV-cytopathic effects [211], constitute critical differences compared to CD4+ T cell reservoirs that constitute additional challenges to achieve a complete cure.

A recent study by Hany et al. analyzed how different LRAs (bryostatin-1, JQ1 and romidepsin) affected human monocyte-derived macrophages [212]. They observed that the treatment with LRAs decreased macrophage susceptibility to HIV-1 infection. Furthermore, bryostatin-1 and romidepsin resulted in downregulation of CD4 and CCR5 receptors, respectively, that was accompanied by a reduction of R5 tropic virus infection. HIV-1 replication was mainly regulated by receptor modulation via bryostatin-1, while romidepsin effects rely on upregulation of SAMHD1 activity. However, these results conflict with a previous study by Campbell et al., showing that romidepsin did not affect CD4 or CCR5 expression in monocyte-derived macrophages [213].

Studies that evaluated the effect of Histone Deacetylase inhibitors (HDACi) in MDMs in vitro showed induction of an autophagy-dependent degradation of viral particles without altering the initial infection of macrophages [213]. This study showed that HDACi decrease HIV release from macrophages in a dose-dependent manner via degradation of intracellular HIV through the canonical autophagy pathway.

Another study using an in vitro primary human MDM model to study reactivation of HIV-1 transcription was used to evaluate latency modulating therapeutic agents [213]. FACS-sorted GFP–MDMs were cultured in the presence of T20 (enfuvirtide), with or without one of the LRAs: bryostatin, panobinostat or vorinostat, and the proportion of reactivated GFP+ cells was quantified. HIV reactivation was significantly increased in MDMs treated with bryostatin and vorinostat. Panobinostat did not significantly alter HIV reactivation in this model. However, LRA-induced reactivation, relative to spontaneous reactivation, differed between donors, suggesting variability in the susceptibility of the individual latent reservoirs to latency modulation [108]. These results suggest that similar to the lymphocyte reservoir, the heterogeneity of monocyte/macrophage subpopulations may have distinct susceptibility to reactivation.

Specific challenges associated with eradication of brain reservoirs have been recently reviewed and we discussed them briefly here [210,214]. Current LRAs that have progressed to clinical evaluation for an HIV cure have mostly been used against cancers and therefore, the information about neurotoxicity and BBB penetrance is available [210]. Overall, the administration of HDACi to PLWH was well tolerated and adverse events were mostly categorized as grade 1 or 2 with the exception of one grade 3 reported in a recent clinical trial [215,216,217,218]. One pilot study assessed the potential neurotoxicity of HDACi, panobinostat, showing that its administration did not induce adverse effects in the CNS [216]. However, the lack of detection of CSF HIV RNA, indicated that panobinostat may also have low BBB penetrance and therefore its utility for targeting CNS reservoirs may be limited. Increasing evidence from basic and preclinical studies demonstrates the need for careful examination of the neurotoxic effects of LRAs and their capacity to induce latency reversal both in lymphoid and myeloid cells [219,220,221,222,223].

The use of toll-like receptor (TLR) agonists as an additional class of LRA has been recently reviewed [224]. Due to their potential dual capacity to reactivate viral latency and induce immune responses, they constitute an attractive area for research. Their capacity to reverse latency has been demonstrated in in vitro studies, animal models and clinical trials. Their role as LRA was previously shown in HIV-infected monocytes/macrophages [224]. In latently infected monocytic cell lines, HIV replication was activated upon treatment with TLR-2, TLR-3 (Poly-I:C), TLR-9 (CpG) and TLR-7/8 (R-848) agonists [225,226,227,228,229]. LPS, a TLR-4 agonist, has also been shown to reactivate latent HIV macrophages from urethra of ART suppressed patients [82]. TLR-2 agonist molecules derived from *Mycobacterium Tuberculosis* have also been shown to reactivate HIV in latency models of microglia [106]. In the humanized mouse and Rhesus macaque models, treatment with (Poly-I:C) and TLR-7 agonist (GS-9620) has demonstrated a reduction in HIV reservoirs in numerous studies [230,231,232,233,234,235]. TLR-7 agonist (GS-9620) and TLR9 (MGN1703) have reached clinical testing in PLWH, although the specific effect on myeloid reservoirs has not yet been evaluated [236,237,238].

## 11. Concluding Remarks

As part of the “kill” portion of the shock and kill strategy, most studies of CD8+ cytotoxic T lymphocytes (CTLs) targeting HIV reservoirs have focused on CD4+ T cells, with few exceptions of targeting macrophage reservoirs. There are studies showing HIV/SIV-infected macrophages are resistant to CTL-mediated killing, even though they can effectively target CD4+ T cells [239,240,241]. This might be due to intrinsic difference in macrophages and CD4+ T cells. Macrophage killing was more dependent on the granzyme B and caspase-3 pathway, which were different from CD4+ T cell killing [239].

Other cytotoxic effector cells are also being investigated for killing HIV-infected macrophages. Natural killer cells were shown to have less effective cytolytic and ADCC response against HIV-infected macrophages compared to CD4+ T cells [242]. We have previously shown that another cytotoxic effector cell, gamma delta (γδ) T cells, have potentials for eliminating persistent HIV in lymphocyte reservoirs [243,244,245]. Our lab is currently exploring the ability of these cells to target non-T cell reservoirs while exploring the development of an immunotherapy for HIV cure [245].

In summary, a complete HIV cure will not happen without elimination of all reservoirs of persistent HIV. Therefore, it is critical to further characterize, investigate and analyze the impact of LRAs on myeloid populations, their susceptibility to reactivation and the source of viral rebound, and killing by effector cells.

## Data Availability

Not applicable.

## References

[1] Chun T.W., Carruth L., Finzi D., Shen X., DiGiuseppe J.A., Taylor H., Hermankova M., Chadwick K., Margolick J., Quinn T.C. (1997). Quantification of latent tissue reservoirs and total body viral load in HIV-1 infection. Nature.

[2] Finzi D., Blankson J., Siliciano J.D., Margolick J.B., Chadwick K., Pierson T., Smith K., Lisziewicz J., Lori F., Flexner C. (1999). Latent infection of CD4+ T cells provides a mechanism for lifelong persistence of HIV-1, even in patients on effective combination therapy. Nat. Med..

[3] Wong J.K., Hezareh M., Gunthard H.F., Havlir D.V., Ignacio C.C., Spina C.A., Richman D.D. (1997). Recovery of replication-competent HIV despite prolonged suppression of plasma viremia. Science.

[4] Archin N.M., Sung J.M., Garrido C., Soriano-Sarabia N., Margolis D.M. (2014). Eradicating HIV-1 infection: Seeking to clear a persistent pathogen. Nat. Rev. Microbiol..

[5] Siliciano J.D., Kajdas J., Finzi D., Quinn T.C., Chadwick K., Margolick J.B., Kovacs C., Gange S.J., Siliciano R.F. (2003). Long-term follow-up studies confirm the stability of the latent reservoir for HIV-1 in resting CD4+ T cells. Nat. Med..

[6] Crooks A.M., Bateson R., Cope A.B., Dahl N.P., Griggs M.K., Kuruc J.D., Gay C.L., Eron J.J., Margolis D.M., Bosch R.J. (2015). Precise Quantitation of the Latent HIV-1 Reservoir: Implications for Eradication Strategies. J. Infect. Dis..

[7] Chomont N., El-Far M., Ancuta P., Trautmann L., Procopio F.A., Yassine-Diab B., Boucher G., Boulassel M.R., Ghattas G., Brenchley J.M. (2009). HIV reservoir size and persistence are driven by T cell survival and homeostatic proliferation. Nat. Med..

[8] Buzon M.J., Sun H., Li C., Shaw A., Seiss K., Ouyang Z., Martin-Gayo E., Leng J., Henrich T.J., Li J.Z. (2014). HIV-1 persistence in CD4+ T cells with stem cell-like properties. Nat. Med..

[9] Soriano-Sarabia N., Bateson R.E., Dahl N.P., Crooks A.M., Kuruc J.D., Margolis D.M., Archin N.M. (2014). Quantitation of replication-competent HIV-1 in populations of resting CD4+ T cells. J. Virol..

[10] Venanzi Rullo E., Pinzone M.R., Cannon L., Weissman S., Ceccarelli M., Zurakowski R., Nunnari G., O’Doherty U. (2020). Persistence of an intact HIV reservoir in phenotypically naive T cells. JCI Insight.

[11] Grau-Expósito J., Luque-Ballesteros L., Navarro J., Curran A., Burgos J., Ribera E., Torrella A., Planas B., Badía R., Martin-Castillo M. (2019). Latency reversal agents affect differently the latent reservoir present in distinct CD4+ T subpopulations. PLoS Pathog..

[12] Reeves D.B., Duke E.R., Wagner T.A., Palmer S.E., Spivak A.M., Schiffer J.T. (2018). A majority of HIV persistence during antiretroviral therapy is due to infected cell proliferation. Nat. Commun..

[13] Cohn L.B., Chomont N., Deeks S.G. (2020). The Biology of the HIV-1 Latent Reservoir and Implications for Cure Strategies. Cell Host Microbe.

[14] von Stockenstrom S., Odevall L., Lee E., Sinclair E., Bacchetti P., Killian M., Epling L., Shao W., Hoh R., Ho T. (2015). Longitudinal Genetic Characterization Reveals That Cell Proliferation Maintains a Persistent HIV Type 1 DNA Pool During Effective HIV Therapy. J. Infect. Dis..

[15] Buzón M.J., Massanella M., Llibre J.M., Esteve A., Dahl V., Puertas M.C., Gatell J.M., Domingo P., Paredes R., Sharkey M. (2010). HIV-1 replication and immune dynamics are affected by raltegravir intensification of HAART-suppressed subjects. Nat. Med..

[16] Fletcher C.V., Staskus K., Wietgrefe S.W., Rothenberger M., Reilly C., Chipman J.G., Beilman G.J., Khoruts A., Thorkelson A., Schmidt T.E. (2014). Persistent HIV-1 replication is associated with lower antiretroviral drug concentrations in lymphatic tissues. Proc. Natl. Acad. Sci. USA.

[17] Lorenzo-Redondo R., Fryer H.R., Bedford T., Kim E.Y., Archer J., Pond S.L.K., Chung Y.S., Penugonda S., Chipman J., Fletcher C.V. (2016). Persistent HIV-1 replication maintains the tissue reservoir during therapy. Nature.

[18] Deeks S.G., Verdin E., McCune J.M. (2012). Immunosenescence and HIV. Curr. Opin. Immunol..

[19] Paiardini M., Müller-Trutwin M. (2013). HIV-associated chronic immune activation. Immunol. Rev..

[20] Vantourout P., Hayday A. (2013). Six-of-the-best: Unique contributions of gammadelta T cells to immunology. Nat. Rev. Immunol..

[21] James K.S., Trumble I., Clohosey M.L., Moeser M., Roan N.R., Adimora A.A., Joseph S.B., Archin N.M., Hudgens M., Soriano-Sarabia N. (2020). Measuring the contribution of γδ T cells to the persistent HIV reservoir. Aids.

[22] Soriano-Sarabia N., Archin N.M., Bateson R., Dahl N.P., Crooks A.M., Kuruc J.D., Garrido C., Margolis D.M. (2015). Peripheral Vgamma9Vdelta2 T Cells Are a Novel Reservoir of Latent HIV Infection. PLoS Pathog..

[23] Gabrilovich D.I., Nagaraj S. (2009). Myeloid-derived suppressor cells as regulators of the immune system. Nat. Rev. Immunol..

[24] Ma T., Renz B.W., Ilmer M., Koch D., Yang Y., Werner J., Bazhin A.V. (2022). Myeloid-Derived Suppressor Cells in Solid Tumors. Cells.

[25] Stegelmeier A.A., van Vloten J.P., Mould R.C., Klafuric E.M., Minott J.A., Wootton S.K., Bridle B.W., Karimi K. (2019). Myeloid Cells during Viral Infections and Inflammation. Viruses.

[26] Summers C., Rankin S.M., Condliffe A.M., Singh N., Peters A.M., Chilvers E.R. (2010). Neutrophil kinetics in health and disease. Trends Immunol..

[27] Murray P.J., Wynn T.A. (2011). Protective and pathogenic functions of macrophage subsets. Nat. Rev. Immunol..

[28] Davies L.C., Jenkins S.J., Allen J.E., Taylor P.R. (2013). Tissue-resident macrophages. Nat. Immunol..

[29] Engblom C., Pfirschke C., Pittet M.J. (2016). The role of myeloid cells in cancer therapies. Nat. Rev. Cancer.

[30] Yona S., Gordon S. (2015). From the Reticuloendothelial to Mononuclear Phagocyte System—The Unaccounted Years. Front. Immunol..

[31] Ginhoux F., Guilliams M. (2016). Tissue-Resident Macrophage Ontogeny and Homeostasis. Immunity.

[32] Tober J., Koniski A., McGrath K.E., Vemishetti R., Emerson R., de Mesy-Bentley K.K., Waugh R., Palis J. (2007). The megakaryocyte lineage originates from hemangioblast precursors and is an integral component both of primitive and of definitive hematopoiesis. Blood.

[33] Samokhvalov I.M., Samokhvalova N.I., Nishikawa S. (2007). Cell tracing shows the contribution of the yolk sac to adult haematopoiesis. Nature.

[34] Hashimoto D., Chow A., Noizat C., Teo P., Beasley M.B., Leboeuf M., Becker C.D., See P., Price J., Lucas D. (2013). Tissue-resident macrophages self-maintain locally throughout adult life with minimal contribution from circulating monocytes. Immunity.

[35] Röszer T. (2018). Understanding the Biology of Self-Renewing Macrophages. Cells.

[36] Williams J.W., Giannarelli C., Rahman A., Randolph G.J., Kovacic J.C. (2018). Macrophage Biology, Classification, and Phenotype in Cardiovascular Disease: JACC Macrophage in CVD Series (Part 1). J. Am. Coll. Cardiol..

[37] Stremmel C., Schuchert R., Wagner F., Thaler R., Weinberger T., Pick R., Mass E., Ishikawa-Ankerhold H.C., Margraf A., Hutter S. (2018). Yolk sac macrophage progenitors traffic to the embryo during defined stages of development. Nat. Commun..

[38] Bain C.C., Schridde A. (2018). Origin, Differentiation, and Function of Intestinal Macrophages. Front. Immunol..

[39] Hu G., Christman J.W. (2019). Editorial: Alveolar Macrophages in Lung Inflammation and Resolution. Front. Immunol..

[40] Wong K.L., Tai J.J., Wong W.C., Han H., Sem X., Yeap W.H., Kourilsky P., Wong S.C. (2011). Gene expression profiling reveals the defining features of the classical, intermediate, and nonclassical human monocyte subsets. Blood.

[41] Zawada A.M., Rogacev K.S., Rotter B., Winter P., Marell R.R., Fliser D., Heine G.H. (2011). SuperSAGE evidence for CD14++CD16+ monocytes as a third monocyte subset. Blood.

[42] Schmidl C., Renner K., Peter K., Eder R., Lassmann T., Balwierz P.J., Itoh M., Nagao-Sato S., Kawaji H., Carninci P. (2014). Transcription and enhancer profiling in human monocyte subsets. Blood.

[43] Merah-Mourah F., Cohen S.O., Charron D., Mooney N., Haziot A. (2020). Identification of Novel Human Monocyte Subsets and Evidence for Phenotypic Groups Defined by Interindividual Variations of Expression of Adhesion Molecules. Sci. Rep..

[44] Gren S.T., Rasmussen T.B., Janciauskiene S., Håkansson K., Gerwien J.G., Grip O. (2015). A Single-Cell Gene-Expression Profile Reveals Inter-Cellular Heterogeneity within Human Monocyte Subsets. PLoS ONE.

[45] Patel V.K., Williams H., Li S.C.H., Fletcher J.P., Medbury H.J. (2017). Monocyte inflammatory profile is specific for individuals and associated with altered blood lipid levels. Atherosclerosis.

[46] Puissant-Lubrano B., Apoil P.A., Guedj K., Congy-Jolivet N., Roubinet F., Guyonnet S., Sourdet S., Nourhashemi F., Blancher A. (2018). Distinct effect of age, sex, and CMV seropositivity on dendritic cells and monocytes in human blood. Immunol. Cell. Biol..

[47] Narasimhan P.B., Marcovecchio P., Hamers A.A.J., Hedrick C.C. (2019). Nonclassical Monocytes in Health and Disease. Annu. Rev. Immunol..

[48] Kapellos T.S., Bonaguro L., Gemünd I., Reusch N., Saglam A., Hinkley E.R., Schultze J.L. (2019). Human Monocyte Subsets and Phenotypes in Major Chronic Inflammatory Diseases. Front. Immunol..

[49] Yam A.O., Chtanova T. (2019). The Ins and Outs of Chemokine-Mediated Immune Cell Trafficking in Skin Cancer. Front. Immunol..

[50] McGovern K.E., Wilson E.H. (2013). Role of Chemokines and Trafficking of Immune Cells in Parasitic Infections. Curr. Immunol. Rev..

[51] Fei L., Ren X., Yu H., Zhan Y. (2021). Targeting the CCL2/CCR2 Axis in Cancer Immunotherapy: One Stone, Three Birds?. Front. Immunol..

[52] Jin J., Lin J., Xu A., Lou J., Qian C., Li X., Wang Y., Yu W., Tao H. (2021). CCL2: An Important Mediator Between Tumor Cells and Host Cells in Tumor Microenvironment. Front. Oncol..

[53] Ancuta P., Rao R., Moses A., Mehle A., Shaw S.K., Luscinskas F.W., Gabuzda D. (2003). Fractalkine preferentially mediates arrest and migration of CD16+ monocytes. J. Exp. Med..

[54] Sandblad K.G., Jones P., Kostalla M.J., Linton L., Glise H., Winqvist O. (2015). Chemokine receptor expression on monocytes from healthy individuals. Clin. Immunol..

[55] Okabe Y., Medzhitov R. (2016). Tissue biology perspective on macrophages. Nat. Immunol..

[56] Bene K., Halasz L., Nagy L. (2021). Transcriptional repression shapes the identity and function of tissue macrophages. FEBS Open Bio.

[57] Merad M., Manz M.G., Karsunky H., Wagers A., Peters W., Charo I., Weissman I.L., Cyster J.G., Engleman E.G. (2002). Langerhans cells renew in the skin throughout life under steady-state conditions. Nat. Immunol..

[58] Ajami B., Bennett J.L., Krieger C., Tetzlaff W., Rossi F.M. (2007). Local self-renewal can sustain CNS microglia maintenance and function throughout adult life. Nat. Neurosci..

[59] Schulz C., Gomez Perdiguero E., Chorro L., Szabo-Rogers H., Cagnard N., Kierdorf K., Prinz M., Wu B., Jacobsen S.E., Pollard J.W. (2012). A lineage of myeloid cells independent of Myb and hematopoietic stem cells. Science.

[60] Yona S., Kim K.W., Wolf Y., Mildner A., Varol D., Breker M., Strauss-Ayali D., Viukov S., Guilliams M., Misharin A. (2013). Fate mapping reveals origins and dynamics of monocytes and tissue macrophages under homeostasis. Immunity.

[61] Koenig S., Gendelman H.E., Orenstein J.M., Dal Canto M.C., Pezeshkpour G.H., Yungbluth M., Janotta F., Aksamit A., Martin M.A., Fauci A.S. (1986). Detection of AIDS virus in macrophages in brain tissue from AIDS patients with encephalopathy. Science.

[62] Koppensteiner H., Brack-Werner R., Schindler M. (2012). Macrophages and their relevance in Human Immunodeficiency Virus Type I infection. Retrovirology.

[63] Veenhuis R.T., Abreu C.M., Shirk E.N., Gama L., Clements J.E. (2021). HIV replication and latency in monocytes and macrophages. Semin. Immunol..

[64] Perelson A.S., Neumann A.U., Markowitz M., Leonard J.M., Ho D.D. (1996). HIV-1 dynamics in vivo: Virion clearance rate, infected cell life-span, and viral generation time. Science.

[65] Bergamaschi A., Pancino G. (2010). Host hindrance to HIV-1 replication in monocytes and macrophages. Retrovirology.

[66] Duncan C.J., Sattentau Q.J. (2011). Viral determinants of HIV-1 macrophage tropism. Viruses.

[67] Gobeil L.A., Lodge R., Tremblay M.J. (2012). Differential HIV-1 endocytosis and susceptibility to virus infection in human macrophages correlate with cell activation status. J. Virol..

[68] Patel A.A., Zhang Y., Fullerton J.N., Boelen L., Rongvaux A., Maini A.A., Bigley V., Flavell R.A., Gilroy D.W., Asquith B. (2017). The fate and lifespan of human monocyte subsets in steady state and systemic inflammation. J. Exp. Med..

[69] Cattin A., Wiche Salinas T.R., Gosselin A., Planas D., Shacklett B., Cohen E.A., Ghali M.P., Routy J.P., Ancuta P. (2019). HIV-1 is rarely detected in blood and colon myeloid cells during viral-suppressive antiretroviral therapy. AIDS.

[70] Williams D.W., Veenstra M., Gaskill P.J., Morgello S., Calderon T.M., Berman J.W. (2014). Monocytes mediate HIV neuropathogenesis: Mechanisms that contribute to HIV associated neurocognitive disorders. Curr. HIV Res..

[71] Ellery P.J., Tippett E., Chiu Y.L., Paukovics G., Cameron P.U., Solomon A., Lewin S.R., Gorry P.R., Jaworowski A., Greene W.C. (2007). The CD16+ monocyte subset is more permissive to infection and preferentially harbors HIV-1 in vivo. J. Immunol..

[72] Jaworowski A., Kamwendo D.D., Ellery P., Sonza S., Mwapasa V., Tadesse E., Molyneux M.E., Rogerson S.J., Meshnick S.R., Crowe S.M. (2007). CD16+ monocyte subset preferentially harbors HIV-1 and is expanded in pregnant Malawian women with Plasmodium falciparum malaria and HIV-1 infection. J. Infect. Dis..

[73] Peng G., Greenwell-Wild T., Nares S., Jin W., Lei K.J., Rangel Z.G., Munson P.J., Wahl S.M. (2007). Myeloid differentiation and susceptibility to HIV-1 are linked to APOBEC3 expression. Blood.

[74] Laguette N., Sobhian B., Casartelli N., Ringeard M., Chable-Bessia C., Segeral E., Yatim A., Emiliani S., Schwartz O., Benkirane M. (2011). SAMHD1 is the dendritic- and myeloid-cell-specific HIV-1 restriction factor counteracted by Vpx. Nature.

[75] Lahouassa H., Daddacha W., Hofmann H., Ayinde D., Logue E.C., Dragin L., Bloch N., Maudet C., Bertrand M., Gramberg T. (2012). SAMHD1 restricts the replication of human immunodeficiency virus type 1 by depleting the intracellular pool of deoxynucleoside triphosphates. Nat. Immunol..

[76] Zhu T., Muthui D., Holte S., Nickle D., Feng F., Brodie S., Hwangbo Y., Mullins J.I., Corey L. (2002). Evidence for human immunodeficiency virus type 1 replication in vivo in CD14(+) monocytes and its potential role as a source of virus in patients on highly active antiretroviral therapy. J. Virol..

[77] Calcaterra S., Cappiello G., Di Caro A., Garbuglia A.R., Benedetto A. (2001). Comparative analysis of total and integrated HIV-1 DNA in peripheral CD4 lymphocytes and monocytes after long treatment with HAART. J. Infect..

[78] Lambotte O., Taoufik Y., de Goer M.G., Wallon C., Goujard C., Delfraissy J.F. (2000). Detection of infectious HIV in circulating monocytes from patients on prolonged highly active antiretroviral therapy. J. Acquir. Immune Defic. Syndr..

[79] Andrade V.M., Mavian C., Babic D., Cordeiro T., Sharkey M., Barrios L., Brander C., Martinez-Picado J., Dalmau J., Llano A. (2020). A minor population of macrophage-tropic HIV-1 variants is identified in recrudescing viremia following analytic treatment interruption. Proc. Natl. Acad. Sci. USA.

[80] Shaw G.M., Hunter E. (2012). HIV transmission. Cold Spring Harb. Perspect. Med..

[81] Zalar A., Figueroa M.I., Ruibal-Ares B., Bare P., Cahn P., de Bracco M.M., Belmonte L. (2010). Macrophage HIV-1 infection in duodenal tissue of patients on long term HAART. Antivir. Res..

[82] Ganor Y., Real F., Sennepin A., Dutertre C.A., Prevedel L., Xu L., Tudor D., Charmeteau B., Couedel-Courteille A., Marion S. (2019). HIV-1 reservoirs in urethral macrophages of patients under suppressive antiretroviral therapy. Nat. Microbiol..

[83] Matusali G., Dereuddre-Bosquet N., Le Tortorec A., Moreau M., Satie A.P., Mahe D., Roumaud P., Bourry O., Sylla N., Bernard-Stoecklin S. (2015). Detection of Simian Immunodeficiency Virus in Semen, Urethra, and Male Reproductive Organs during Efficient Highly Active Antiretroviral Therapy. J. Virol..

[84] Hufert F.T., Schmitz J., Schreiber M., Schmitz H., Racz P., von Laer D.D. (1993). Human Kupffer cells infected with HIV-1 in vivo. J. Acquir. Immune Defic. Syndr. (1988).

[85] Ahsan M.H., Gill A.F., Alvarez X., Lackner A.A., Veazey R.S. (2013). Kinetics of liver macrophages (Kupffer cells) in SIV-infected macaques. Virology.

[86] Cribbs S.K., Lennox J., Caliendo A.M., Brown L.A., Guidot D.M. (2015). Healthy HIV-1-infected individuals on highly active antiretroviral therapy harbor HIV-1 in their alveolar macrophages. AIDS Res. Hum. Retrovir..

[87] DiNapoli S.R., Ortiz A.M., Wu F., Matsuda K., Twigg H.L., Hirsch V.M., Knox K., Brenchley J.M. (2017). Tissue-resident macrophages can contain replication-competent virus in antiretroviral-naive, SIV-infected Asian macaques. JCI Insight.

[88] Wong M.E., Jaworowski A., Hearps A.C. (2019). The HIV Reservoir in Monocytes and Macrophages. Front. Immunol..

[89] Jambo K.C., Banda D.H., Kankwatira A.M., Sukumar N., Allain T.J., Heyderman R.S., Russell D.G., Mwandumba H.C. (2014). Small alveolar macrophages are infected preferentially by HIV and exhibit impaired phagocytic function. Mucosal Immunol..

[90] Ganor Y., Zhou Z., Tudor D., Schmitt A., Vacher-Lavenu M.C., Gibault L., Thiounn N., Tomasini J., Wolf J.P., Bomsel M. (2010). Within 1 h, HIV-1 uses viral synapses to enter efficiently the inner, but not outer, foreskin mucosa and engages Langerhans-T cell conjugates. Mucosal Immunol..

[91] Zhou Z., Barry de Longchamps N., Schmitt A., Zerbib M., Vacher-Lavenu M.C., Bomsel M., Ganor Y. (2011). HIV-1 efficient entry in inner foreskin is mediated by elevated CCL5/RANTES that recruits T cells and fuels conjugate formation with Langerhans cells. PLoS Pathog..

[92] Ganor Y., Zhou Z., Bodo J., Tudor D., Leibowitch J., Mathez D., Schmitt A., Vacher-Lavenu M.C., Revol M., Bomsel M. (2013). The adult penile urethra is a novel entry site for HIV-1 that preferentially targets resident urethral macrophages. Mucosal Immunol..

[93] de Witte L., Nabatov A., Pion M., Fluitsma D., de Jong M.A., de Gruijl T., Piguet V., van Kooyk Y., Geijtenbeek T.B. (2007). Langerin is a natural barrier to HIV-1 transmission by Langerhans cells. Nat. Med..

[94] Kalter D.C., Greenhouse J.J., Orenstein J.M., Schnittman S.M., Gendelman H.E., Meltzer M.S. (1991). Epidermal Langerhans cells are not principal reservoirs of virus in HIV disease. J. Immunol..

[95] Bhoopat L., Rithaporn T.S., Khunamornpong S., Bhoopat T., Taylor C.R., Thorner P.S. (2006). Cell reservoirs in lymph nodes infected with HIV-1 subtype E differ from subtype B: Identification by combined in situ polymerase chain reaction and immunohistochemistry. Mod. Pathol..

[96] Pena-Cruz V., Agosto L.M., Akiyama H., Olson A., Moreau Y., Larrieux J.R., Henderson A., Gummuluru S., Sagar M. (2018). HIV-1 replicates and persists in vaginal epithelial dendritic cells. J. Clin. Investig..

[97] Cassol E., Alfano M., Biswas P., Poli G. (2006). Monocyte-derived macrophages and myeloid cell lines as targets of HIV-1 replication and persistence. J. Leukoc. Biol..

[98] Folks T.M., Justement J., Kinter A., Schnittman S., Orenstein J., Poli G., Fauci A.S. (1988). Characterization of a promonocyte clone chronically infected with HIV and inducible by 13-phorbol-12-myristate acetate. J. Immunol..

[99] Vicenzi E., Biswas P., Mengozzi M., Poli G. (1997). Role of pro-inflammatory cytokines and beta-chemokines in controlling HIV replication. J. Leukoc. Biol..

[100] Poli G., Kinter A., Justement J.S., Kehrl J.H., Bressler P., Stanley S., Fauci A.S. (1990). Tumor necrosis factor alpha functions in an autocrine manner in the induction of human immunodeficiency virus expression. Proc. Natl. Acad. Sci. USA.

[101] Tsuchiya S., Yamabe M., Yamaguchi Y., Kobayashi Y., Konno T., Tada K. (1980). Establishment and characterization of a human acute monocytic leukemia cell line (THP-1). Int. J. Cancer.

[102] Kohro T., Tanaka T., Murakami T., Wada Y., Aburatani H., Hamakubo T., Kodama T. (2004). A comparison of differences in the gene expression profiles of phorbol 12-myristate 13-acetate differentiated THP-1 cells and human monocyte-derived macrophage. J. Atheroscler. Thromb..

[103] Konopka K., Pretzer E., Plowman B., Duzgunes N. (1993). Long-term noncytopathic productive infection of the human monocytic leukemia cell line THP-1 by human immunodeficiency virus type 1 (HIV-1IIIB). Virology.

[104] Mikovits J.A., Raziuddin, Gonda M., Ruta M., Lohrey N.C., Kung H.F., Ruscetti F.W. (1990). Negative regulation of human immune deficiency virus replication in monocytes. Distinctions between restricted and latent expression in THP-1 cells. J. Exp. Med..

[105] Mayers D.L., Mikovits J.A., Joshi B., Hewlett I.K., Estrada J.S., Wolfe A.D., Garcia G.E., Doctor B.P., Burke D.S., Gordon R.K. (1995). Anti-human immunodeficiency virus 1 (HIV-1) activities of 3-deazaadenosine analogs: Increased potency against 3’-azido-3’-deoxythymidine-resistant HIV-1 strains. Proc. Natl. Acad. Sci. USA.

[106] Alvarez-Carbonell D., Garcia-Mesa Y., Milne S., Das B., Dobrowolski C., Rojas R., Karn J. (2017). Toll-like receptor 3 activation selectively reverses HIV latency in microglial cells. Retrovirology.

[107] Alvarez-Carbonell D., Ye F., Ramanath N., Garcia-Mesa Y., Knapp P.E., Hauser K.F., Karn J. (2019). Cross-talk between microglia and neurons regulates HIV latency. PLoS Pathog..

[108] Wong M.E., Johnson C.J., Hearps A.C., Jaworowski A. (2021). Development of a Novel In Vitro Primary Human Monocyte-Derived Macrophage Model To Study Reactivation of HIV-1 Transcription. J. Virol..

[109] Mantovani A., Sica A., Sozzani S., Allavena P., Vecchi A.A., Locati M. (2004). The chemokine system in diverse forms of macrophage activation and polarization. Trends Immunol..

[110] Durafourt B.A., Moore C.S., Zammit D.A., Johnson T.A., Zaguia F., Guiot M.C., Bar-Or A., Antel J.P. (2012). Comparison of polarization properties of human adult microglia and blood-derived macrophages. Glia.

[111] Vogel D.Y., Glim J.E., Stavenuiter A.W., Breur M., Heijnen P., Amor S., Dijkstra C.D., Beelen R.H. (2014). Human macrophage polarization in vitro: Maturation and activation methods compared. Immunobiology.

[112] Graziano F., Vicenzi E., Poli G. (2016). Plastic restriction of HIV-1 replication in human macrophages derived from M1/M2 polarized monocytes. J. Leukoc. Biol..

[113] Atri C., Guerfali F.Z., Laouini D. (2018). Role of Human Macrophage Polarization in Inflammation during Infectious Diseases. Int. J. Mol. Sci..

[114] Cassol E., Cassetta L., Rizzi C., Alfano M., Poli G. (2009). M1 and M2a polarization of human monocyte-derived macrophages inhibits HIV-1 replication by distinct mechanisms. J. Immunol..

[115] Cassol E., Cassetta L., Alfano M., Poli G. (2010). Macrophage polarization and HIV-1 infection. J. Leukoc. Biol..

[116] Brown A., Zhang H., Lopez P., Pardo C.A., Gartner S. (2006). In vitro modeling of the HIV-macrophage reservoir. J. Leukoc. Biol..

[117] Agarwal Y., Beatty C., Biradar S., Castronova I., Ho S., Melody K., Bility M.T. (2020). Moving beyond the mousetrap: Current and emerging humanized mouse and rat models for investigating prevention and cure strategies against HIV infection and associated pathologies. Retrovirology.

[118] Marsden M.D. (2020). Benefits and limitations of humanized mice in HIV persistence studies. Retrovirology.

[119] Terahara K., Iwabuchi R., Tsunetsugu-Yokota Y. (2021). Perspectives on Non-BLT Humanized Mouse Models for Studying HIV Pathogenesis and Therapy. Viruses.

[120] Kim K.C., Choi B.S., Kim K.C., Park K.H., Lee H.J., Cho Y.K., Kim S.I., Kim S.S., Oh Y.K., Kim Y.B. (2016). A Simple Mouse Model for the Study of Human Immunodeficiency Virus. AIDS Res. Hum. Retrovir..

[121] Ali N., Flutter B., Sanchez Rodriguez R., Sharif-Paghaleh E., Barber L.D., Lombardi G., Nestle F.O. (2012). Xenogeneic graft-versus-host-disease in NOD-scid IL-2Rgammanull mice display a T-effector memory phenotype. PLoS ONE.

[122] Poluektova L.Y., Munn D.H., Persidsky Y., Gendelman H.E. (2002). Generation of cytotoxic T cells against virus-infected human brain macrophages in a murine model of HIV-1 encephalitis. J. Immunol..

[123] Denton P.W., Garcia J.V. (2011). Humanized mouse models of HIV infection. AIDS Rev..

[124] Arainga M., Edagwa B., Mosley R.L., Poluektova L.Y., Gorantla S., Gendelman H.E. (2017). A mature macrophage is a principal HIV-1 cellular reservoir in humanized mice after treatment with long acting antiretroviral therapy. Retrovirology.

[125] Arainga M., Su H., Poluektova L.Y., Gorantla S., Gendelman H.E. (2016). HIV-1 cellular and tissue replication patterns in infected humanized mice. Sci. Rep..

[126] Li Y., Masse-Ranson G., Garcia Z., Bruel T., Kok A., Strick-Marchand H., Jouvion G., Serafini N., Lim A.I., Dusseaux M. (2018). A human immune system mouse model with robust lymph node development. Nat. Methods.

[127] Watanabe Y., Takahashi T., Okajima A., Shiokawa M., Ishii N., Katano I., Ito R., Ito M., Minegishi M., Minegishi N. (2009). The analysis of the functions of human B and T cells in humanized NOD/shi-scid/gammac(null) (NOG) mice (hu-HSC NOG mice). Int. Immunol..

[128] Shultz L.D., Saito Y., Najima Y., Tanaka S., Ochi T., Tomizawa M., Doi T., Sone A., Suzuki N., Fujiwara H. (2010). Generation of functional human T-cell subsets with HLA-restricted immune responses in HLA class I expressing NOD/SCID/IL2r gamma(null) humanized mice. Proc. Natl. Acad. Sci. USA.

[129] Berges B.K., Akkina S.R., Folkvord J.M., Connick E., Akkina R. (2008). Mucosal transmission of R5 and X4 tropic HIV-1 via vaginal and rectal routes in humanized Rag2^−/−^ gammac^−/−^ (RAG-hu) mice. Virology.

[130] Hofer U., Baenziger S., Heikenwalder M., Schlaepfer E., Gehre N., Regenass S., Brunner T., Speck R.F. (2008). RAG2^−/−^ gamma(c)^−/−^ mice transplanted with CD34+ cells from human cord blood show low levels of intestinal engraftment and are resistant to rectal transmission of human immunodeficiency virus. J. Virol..

[131] Honeycutt J.B., Wahl A., Baker C., Spagnuolo R.A., Foster J., Zakharova O., Wietgrefe S., Caro-Vegas C., Madden V., Sharpe G. (2016). Macrophages sustain HIV replication in vivo independently of T cells. J. Clin. Investig..

[132] Honeycutt J.B., Thayer W.O., Baker C.E., Ribeiro R.M., Lada S.M., Cao Y., Cleary R.A., Hudgens M.G., Richman D.D., Garcia J.V. (2017). HIV persistence in tissue macrophages of humanized myeloid-only mice during antiretroviral therapy. Nat. Med..

[133] Lan P., Tonomura N., Shimizu A., Wang S., Yang Y.G. (2006). Reconstitution of a functional human immune system in immunodeficient mice through combined human fetal thymus/liver and CD34+ cell transplantation. Blood.

[134] Lavender K.J., Pang W.W., Messer R.J., Duley A.K., Race B., Phillips K., Scott D., Peterson K.E., Chan C.K., Dittmer U. (2013). BLT-humanized C57BL/6 Rag2^−/−^gammac^−/−^CD47^−/−^ mice are resistant to GVHD and develop B- and T-cell immunity to HIV infection. Blood.

[135] Denton P.W., Olesen R., Choudhary S.K., Archin N.M., Wahl A., Swanson M.D., Chateau M., Nochi T., Krisko J.F., Spagnuolo R.A. (2012). Generation of HIV latency in humanized BLT mice. J. Virol..

[136] Denton P.W., Estes J.D., Sun Z., Othieno F.A., Wei B.L., Wege A.K., Powell D.A., Payne D., Haase A.T., Garcia J.V. (2008). Antiretroviral pre-exposure prophylaxis prevents vaginal transmission of HIV-1 in humanized BLT mice. PLoS Med..

[137] Olesen R., Wahl A., Denton P.W., Garcia J.V. (2011). Immune reconstitution of the female reproductive tract of humanized BLT mice and their susceptibility to human immunodeficiency virus infection. J. Reprod. Immunol..

[138] Stoddart C.A., Maidji E., Galkina S.A., Kosikova G., Rivera J.M., Moreno M.E., Sloan B., Joshi P., Long B.R. (2011). Superior human leukocyte reconstitution and susceptibility to vaginal HIV transmission in humanized NOD-scid IL-2Rgamma(−/−) (NSG) BLT mice. Virology.

[139] Wahl A., Swanson M.D., Nochi T., Olesen R., Denton P.W., Chateau M., Garcia J.V. (2012). Human breast milk and antiretrovirals dramatically reduce oral HIV-1 transmission in BLT humanized mice. PLoS Pathog..

[140] Sun Z., Denton P.W., Estes J.D., Othieno F.A., Wei B.L., Wege A.K., Melkus M.W., Padgett-Thomas A., Zupancic M., Haase A.T. (2007). Intrarectal transmission, systemic infection, and CD4+ T cell depletion in humanized mice infected with HIV-1. J. Exp. Med..

[141] Lavender K.J., Pace C., Sutter K., Messer R.J., Pouncey D.L., Cummins N.W., Natesampillai S., Zheng J., Goldsmith J., Widera M. (2018). An advanced BLT-humanized mouse model for extended HIV-1 cure studies. AIDS.

[142] Marsden M.D., Kovochich M., Suree N., Shimizu S., Mehta R., Cortado R., Bristol G., An D.S., Zack J.A. (2012). HIV latency in the humanized BLT mouse. J. Virol..

[143] Honeycutt J.B., Liao B., Nixon C.C., Cleary R.A., Thayer W.O., Birath S.L., Swanson M.D., Sheridan P., Zakharova O., Prince F. (2018). T cells establish and maintain CNS viral infection in HIV-infected humanized mice. J. Clin. Investig..

[144] Greenblatt M.B., Vrbanac V., Tivey T., Tsang K., Tager A.M., Aliprantis A.O. (2012). Graft versus host disease in the bone marrow, liver and thymus humanized mouse model. PLoS ONE.

[145] Samal J., Kelly S., Na-Shatal A., Elhakiem A., Das A., Ding M., Sanyal A., Gupta P., Melody K., Roland B. (2018). Human immunodeficiency virus infection induces lymphoid fibrosis in the BM-liver-thymus-spleen humanized mouse model. JCI Insight.

[146] Nixon C.C., Mavigner M., Sampey G.C., Brooks A.D., Spagnuolo R.A., Irlbeck D.M., Mattingly C., Ho P.T., Schoof N., Cammon C.G. (2020). Systemic HIV and SIV latency reversal via non-canonical NF-κB signalling in vivo. Nature.

[147] Terrade G., Huot N., Petitdemange C., Lazzerini M., Orta Resendiz A., Jacquelin B., Muller-Trutwin M. (2021). Interests of the Non-Human Primate Models for HIV Cure Research. Vaccines.

[148] Garcia-Tellez T., Huot N., Ploquin M.J., Rascle P., Jacquelin B., Muller-Trutwin M. (2016). Non-human primates in HIV research: Achievements, limits and alternatives. Infect. Genet. Evol..

[149] Sodora D.L., Gettie A., Miller C.J., Marx P.A. (1998). Vaginal transmission of SIV: Assessing infectivity and hormonal influences in macaques inoculated with cell-free and cell-associated viral stocks. AIDS Res. Hum. Retrovir..

[150] Mir K.D., Mavigner M., Wang C., Paiardini M., Sodora D.L., Chahroudi A.M., Bosinger S.E., Silvestri G. (2015). Reduced Simian Immunodeficiency Virus Replication in Macrophages of Sooty Mangabeys Is Associated with Increased Expression of Host Restriction Factors. J. Virol..

[151] Micci L., Alvarez X., Iriele R.I., Ortiz A.M., Ryan E.S., McGary C.S., Deleage C., McAtee B.B., He T., Apetrei C. (2014). CD4 depletion in SIV-infected macaques results in macrophage and microglia infection with rapid turnover of infected cells. PLoS Pathog..

[152] Moeser M., Nielsen J.R., Joseph S.B. (2020). Macrophage Tropism in Pathogenic HIV-1 and SIV Infections. Viruses.

[153] Nowlin B.T., Burdo T.H., Midkiff C.C., Salemi M., Alvarez X., Williams K.C. (2015). SIV encephalitis lesions are composed of CD163(+) macrophages present in the central nervous system during early SIV infection and SIV-positive macrophages recruited terminally with AIDS. Am. J. Pathol..

[154] Ortiz A.M., Klatt N.R., Li B., Yi Y., Tabb B., Hao X.P., Sternberg L., Lawson B., Carnathan P.M., Cramer E.M. (2011). Depletion of CD4(+) T cells abrogates post-peak decline of viremia in SIV-infected rhesus macaques. J. Clin. Investig..

[155] Abreu C.M., Veenhuis R.T., Avalos C.R., Graham S., Parrilla D.R., Ferreira E.A., Queen S.E., Shirk E.N., Bullock B.T., Li M. (2019). Myeloid and CD4 T Cells Comprise the Latent Reservoir in Antiretroviral Therapy-Suppressed SIVmac251-Infected Macaques. mBio.

[156] Ryzhova E.V., Crino P., Shawver L., Westmoreland S.V., Lackner A.A., Gonzalez-Scarano F. (2002). Simian immunodeficiency virus encephalitis: Analysis of envelope sequences from individual brain multinucleated giant cells and tissue samples. Virology.

[157] Clements J.E., Babas T., Mankowski J.L., Suryanarayana K., Piatak M., Tarwater P.M., Lifson J.D., Zink M.C. (2002). The central nervous system as a reservoir for simian immunodeficiency virus (SIV): Steady-state levels of SIV DNA in brain from acute through asymptomatic infection. J. Infect. Dis..

[158] Zink M.C., Suryanarayana K., Mankowski J.L., Shen A., Piatak M., Spelman J.P., Carter D.L., Adams R.J., Lifson J.D., Clements J.E. (1999). High viral load in the cerebrospinal fluid and brain correlates with severity of simian immunodeficiency virus encephalitis. J. Virol..

[159] Matsuda K., Riddick N.E., Lee C.A., Puryear S.B., Wu F., Lafont B.A.P., Whitted S., Hirsch V.M. (2017). A SIV molecular clone that targets the CNS and induces neuroAIDS in rhesus macaques. PLoS Pathog..

[160] Avalos C.R., Abreu C.M., Queen S.E., Li M., Price S., Shirk E.N., Engle E.L., Forsyth E., Bullock B.T., Mac Gabhann F. (2017). Brain Macrophages in Simian Immunodeficiency Virus-Infected, Antiretroviral-Suppressed Macaques: A Functional Latent Reservoir. mBio.

[161] Avalos C.R., Price S.L., Forsyth E.R., Pin J.N., Shirk E.N., Bullock B.T., Queen S.E., Li M., Gellerup D., O’Connor S.L. (2016). Quantitation of Productively Infected Monocytes and Macrophages of Simian Immunodeficiency Virus-Infected Macaques. J. Virol..

[162] Calantone N., Wu F., Klase Z., Deleage C., Perkins M., Matsuda K., Thompson E.A., Ortiz A.M., Vinton C.L., Ourmanov I. (2014). Tissue myeloid cells in SIV-infected primates acquire viral DNA through phagocytosis of infected T cells. Immunity.

[163] Policicchio B.B., Pandrea I., Apetrei C. (2016). Animal Models for HIV Cure Research. Front. Immunol..

[164] Li Q., Barres B.A. (2018). Microglia and macrophages in brain homeostasis and disease. Nat. Rev. Immunol..

[165] Ginhoux F., Lim S., Hoeffel G., Low D., Huber T. (2013). Origin and differentiation of microglia. Front. Cell. Neurosci..

[166] Ginhoux F., Greter M., Leboeuf M., Nandi S., See P., Gokhan S., Mehler M.F., Conway S.J., Ng L.G., Stanley E.R. (2010). Fate mapping analysis reveals that adult microglia derive from primitive macrophages. Science.

[167] Sheng J., Ruedl C., Karjalainen K. (2015). Most Tissue-Resident Macrophages Except Microglia Are Derived from Fetal Hematopoietic Stem Cells. Immunity.

[168] Nimmerjahn A., Kirchhoff F., Helmchen F. (2005). Resting microglial cells are highly dynamic surveillants of brain parenchyma in vivo. Science.

[169] Aspelund A., Antila S., Proulx S.T., Karlsen T.V., Karaman S., Detmar M., Wiig H., Alitalo K. (2015). A dural lymphatic vascular system that drains brain interstitial fluid and macromolecules. J. Exp. Med..

[170] Louveau A., Smirnov I., Keyes T.J., Eccles J.D., Rouhani S.J., Peske J.D., Derecki N.C., Castle D., Mandell J.W., Lee K.S. (2015). Structural and functional features of central nervous system lymphatic vessels. Nature.

[171] Churchill M.J., Gorry P.R., Cowley D., Lal L., Sonza S., Purcell D.F., Thompson K.A., Gabuzda D., McArthur J.C., Pardo C.A. (2006). Use of laser capture microdissection to detect integrated HIV-1 DNA in macrophages and astrocytes from autopsy brain tissues. J. Neurovirol..

[172] Desplats P., Dumaop W., Smith D., Adame A., Everall I., Letendre S., Ellis R., Cherner M., Grant I., Masliah E. (2013). Molecular and pathologic insights from latent HIV-1 infection in the human brain. Neurology.

[173] Ash M.K., Al-Harthi L., Schneider J.R. (2021). HIV in the Brain: Identifying Viral Reservoirs and Addressing the Challenges of an HIV Cure. Vaccines.

[174] Valcour V., Chalermchai T., Sailasuta N., Marovich M., Lerdlum S., Suttichom D., Suwanwela N.C., Jagodzinski L., Michael N., Spudich S. (2012). Central nervous system viral invasion and inflammation during acute HIV infection. J. Infect. Dis..

[175] Witwer K.W., Gama L., Li M., Bartizal C.M., Queen S.E., Varrone J.J., Brice A.K., Graham D.R., Tarwater P.M., Mankowski J.L. (2009). Coordinated regulation of SIV replication and immune responses in the CNS. PLoS ONE.

[176] Edén A., Fuchs D., Hagberg L., Nilsson S., Spudich S., Svennerholm B., Price R.W., Gisslén M. (2010). HIV-1 viral escape in cerebrospinal fluid of subjects on suppressive antiretroviral treatment. J. Infect. Dis..

[177] Balcom E.F., Roda W.C., Cohen E.A., Li M.Y., Power C. (2019). HIV-1 persistence in the central nervous system: Viral and host determinants during antiretroviral therapy. Curr. Opin. Virol..

[178] Tuttle D.L., Harrison J.K., Anders C., Sleasman J.W., Goodenow M.M. (1998). Expression of CCR5 increases during monocyte differentiation and directly mediates macrophage susceptibility to infection by human immunodeficiency virus type 1. J. Virol..

[179] Shaw T.N., Houston S.A., Wemyss K., Bridgeman H.M., Barbera T.A., Zangerle-Murray T., Strangward P., Ridley A.J.L., Wang P., Tamoutounour S. (2018). Tissue-resident macrophages in the intestine are long lived and defined by Tim-4 and CD4 expression. J. Exp. Med..

[180] Wang X., Ye L., Hou W., Zhou Y., Wang Y.J., Metzger D.S., Ho W.Z. (2009). Cellular microRNA expression correlates with susceptibility of monocytes/macrophages to HIV-1 infection. Blood.

[181] Youssefian T., Drouin A., Masse J.M., Guichard J., Cramer E.M. (2002). Host defense role of platelets: Engulfment of HIV and Staphylococcus aureus occurs in a specific subcellular compartment and is enhanced by platelet activation. Blood.

[182] Banerjee M., Huang Y., Joshi S., Popa G.J., Mendenhall M.D., Wang Q.J., Garvy B.A., Myint T., Whiteheart S.W. (2020). Platelets Endocytose Viral Particles and Are Activated via TLR (Toll-Like Receptor) Signaling. Arter. Thromb Vasc. Biol..

[183] Beck Z., Jagodzinski L.L., Eller M.A., Thelian D., Matyas G.R., Kunz A.N., Alving C.R. (2013). Platelets and erythrocyte-bound platelets bind infectious HIV-1 in plasma of chronically infected patients. PLoS ONE.

[184] Lee T.H., Stromberg R.R., Heitman J.W., Sawyer L., Hanson C.V., Busch M.P. (1998). Distribution of HIV type 1 (HIV-1) in blood components: Detection and significance of high levels of HIV-1 associated with platelets. Transfusion.

[185] Real F., Capron C., Sennepin A., Arrigucci R., Zhu A., Sannier G., Zheng J., Xu L., Masse J.M., Greffe S. (2020). Platelets from HIV-infected individuals on antiretroviral drug therapy with poor CD4(+) T cell recovery can harbor replication-competent HIV despite viral suppression. Sci. Transl. Med..

[186] Simpson S.R., Singh M.V., Dewhurst S., Schifitto G., Maggirwar S.B. (2020). Platelets function as an acute viral reservoir during HIV-1 infection by harboring virus and T-cell complex formation. Blood Adv..

[187] Chaipan C., Soilleux E.J., Simpson P., Hofmann H., Gramberg T., Marzi A., Geier M., Stewart E.A., Eisemann J., Steinkasserer A. (2006). DC-SIGN and CLEC-2 mediate human immunodeficiency virus type 1 capture by platelets. J. Virol..

[188] Park I.W., Wang J.F., Groopman J.E. (1999). Expression and utilization of co-receptors in HIV and simian immunodeficiency virus infection of megakaryocytes. AIDS.

[189] Voulgaropoulou F., Pontow S.E., Ratner L. (2000). Productive infection of CD34+-cell-derived megakaryocytes by X4 and R5 HIV-1 isolates. Virology.

[190] Sato T., Sekine H., Kakuda H., Miura N., Sunohara M., Fuse A. (2000). HIV infection of megakaryocytic cell lines. Leuk. Lymphoma.

[191] d’Arminio Monforte A., Cinque P., Mocroft A., Goebel F.D., Antunes F., Katlama C., Justesen U.S., Vella S., Kirk O., Lundgren J. (2004). Changing incidence of central nervous system diseases in the EuroSIDA cohort. Ann. Neurol..

[192] Sacktor N. (2002). The epidemiology of human immunodeficiency virus-associated neurological disease in the era of highly active antiretroviral therapy. J. Neurovirol..

[193] Saylor D., Dickens A.M., Sacktor N., Haughey N., Slusher B., Pletnikov M., Mankowski J.L., Brown A., Volsky D.J., McArthur J.C. (2016). HIV-associated neurocognitive disorder—Pathogenesis and prospects for treatment. Nat. Rev. Neurol..

[194] Smail R.C., Brew B.J. (2018). HIV-associated neurocognitive disorder. Handb. Clin. Neurol..

[195] Underwood J., Robertson K.R., Winston A. (2015). Could antiretroviral neurotoxicity play a role in the pathogenesis of cognitive impairment in treated HIV disease?. Aids.

[196] Fernandes N., Pulliam L. (2021). Inflammatory Mechanisms and Cascades Contributing to Neurocognitive Impairment in HIV/AIDS. Curr. Top. Behav. Neurosci..

[197] Smith A.S., Ankam S., Farhy C., Fiengo L., Basa R.C.B., Gordon K.L., Martin C.T., Terskikh A.V., Jordan-Sciutto K.L., Price J.H. (2022). High-content analysis and Kinetic Image Cytometry identify toxicity and epigenetic effects of HIV antiretrovirals on human iPSC-neurons and primary neural precursor cells. J. Pharmacol. Toxicol. Methods.

[198] Rawson T., Muir D., Mackie N.E., Garvey L.J., Everitt A., Winston A. (2012). Factors associated with cerebrospinal fluid HIV RNA in HIV infected subjects undergoing lumbar puncture examination in a clinical setting. J. Infect..

[199] Nightingale S., Michael B.D., Fisher M., Winston A., Nelson M., Taylor S., Ustianowski A., Ainsworth J., Gilson R., Haddow L. (2016). CSF/plasma HIV-1 RNA discordance even at low levels is associated with up-regulation of host inflammatory mediators in CSF. Cytokine.

[200] Garvey L.J., Everitt A., Winston A., Mackie N.E., Benzie A. (2009). Detectable cerebrospinal fluid HIV RNA with associated neurological deficits, despite suppression of HIV replication in the plasma compartment. AIDS.

[201] Spudich S., Lollo N., Liegler T., Deeks S.G., Price R.W. (2006). Treatment benefit on cerebrospinal fluid HIV-1 levels in the setting of systemic virological suppression and failure. J. Infect. Dis..

[202] Canestri A., Lescure F.X., Jaureguiberry S., Moulignier A., Amiel C., Marcelin A.G., Peytavin G., Tubiana R., Pialoux G., Katlama C. (2010). Discordance between cerebral spinal fluid and plasma HIV replication in patients with neurological symptoms who are receiving suppressive antiretroviral therapy. Clin. Infect. Dis..

[203] Yilmaz A., Price R.W., Spudich S., Fuchs D., Hagberg L., Gisslén M. (2008). Persistent intrathecal immune activation in HIV-1-infected individuals on antiretroviral therapy. J. Acquir. Immune Defic. Syndr..

[204] Edén A., Price R.W., Spudich S., Fuchs D., Hagberg L., Gisslén M. (2007). Immune activation of the central nervous system is still present after >4 years of effective highly active antiretroviral therapy. J. Infect. Dis..

[205] Young K.G., Maclean S., Dudani R., Krishnan L., Sad S. (2011). CD8+ T cells primed in the periphery provide time-bound immune-surveillance to the central nervous system. J. Immunol..

[206] Smolders J., Remmerswaal E.B., Schuurman K.G., Melief J., van Eden C.G., van Lier R.A., Huitinga I., Hamann J. (2013). Characteristics of differentiated CD8(+) and CD4 (+) T cells present in the human brain. Acta Neuropathol..

[207] Veenstra M., León-Rivera R., Li M., Gama L., Clements J.E., Berman J.W. (2017). Mechanisms of CNS Viral Seeding by HIV(+) CD14(+) CD16(+) Monocytes: Establishment and Reseeding of Viral Reservoirs Contributing to HIV-Associated Neurocognitive Disorders. mBio.

[208] Deeks S.G. (2012). HIV: Shock and kill. Nature.

[209] Barber S.A., Gama L., Dudaronek J.M., Voelker T., Tarwater P.M., Clements J.E. (2006). Mechanism for the establishment of transcriptional HIV latency in the brain in a simian immunodeficiency virus-macaque model. J. Infect. Dis..

[210] Veenhuis R.T., Clements J.E., Gama L. (2019). HIV Eradication Strategies: Implications for the Central Nervous System. Curr. HIV/AIDS Rep..

[211] Paim A.C., Badley A.D., Cummins N.W. (2020). Mechanisms of Human Immunodeficiency Virus-Associated Lymphocyte Regulated Cell Death. AIDS Res. Hum. Retrovir..

[212] Hany L., Turmel M.O., Barat C., Ouellet M., Tremblay M.J. (2022). Bryostatin-1 Decreases HIV-1 Infection and Viral Production in Human Primary Macrophages. J. Virol..

[213] Campbell G.R., Bruckman R.S., Chu Y.L., Spector S.A. (2015). Autophagy induction by histone deacetylase inhibitors inhibits HIV type 1. J. Biol. Chem..

[214] Borrajo A., Svicher V., Salpini R., Pellegrino M., Aquaro S. (2021). Crucial Role of Central Nervous System as a Viral Anatomical Compartment for HIV-1 Infection. Microorganisms.

[215] Archin N.M., Liberty A.L., Kashuba A.D., Choudhary S.K., Kuruc J.D., Crooks A.M., Parker D.C., Anderson E.M., Kearney M.F., Strain M.C. (2012). Administration of vorinostat disrupts HIV-1 latency in patients on antiretroviral therapy. Nature.

[216] Rasmussen T.A., Tolstrup M., Møller H.J., Brinkmann C.R., Olesen R., Erikstrup C., Laursen A.L., Østergaard L., Søgaard O.S. (2015). Activation of latent human immunodeficiency virus by the histone deacetylase inhibitor panobinostat: A pilot study to assess effects on the central nervous system. Open Forum Infect. Dis..

[217] Sogaard O.S., Graversen M.E., Leth S., Olesen R., Brinkmann C.R., Nissen S.K., Kjaer A.S., Schleimann M.H., Denton P.W., Hey-Cunningham W.J. (2015). The Depsipeptide Romidepsin Reverses HIV-1 Latency In Vivo. PLoS Pathog..

[218] McMahon D.K., Zheng L., Cyktor J.C., Aga E., Macatangay B.J., Godfrey C., Para M., Mitsuyasu R.T., Hesselgesser J., Dragavon J. (2021). A Phase 1/2 Randomized, Placebo-Controlled Trial of Romidespin in Persons With HIV-1 on Suppressive Antiretroviral Therapy. J. Infect. Dis..

[219] Proust A., Barat C., Leboeuf M., Drouin J., Tremblay M.J. (2017). Contrasting effect of the latency-reversing agents bryostatin-1 and JQ1 on astrocyte-mediated neuroinflammation and brain neutrophil invasion. J. Neuroinflamm..

[220] Gama L., Abreu C.M., Shirk E.N., Price S.L., Li M., Laird G.M., Pate K.A., Wietgrefe S.W., O’Connor S.L., Pianowski L. (2017). Reactivation of simian immunodeficiency virus reservoirs in the brain of virally suppressed macaques. AIDS.

[221] Caballero R.E., Dong S.X.M., Gajanayaka N., Ali H., Cassol E., Cameron W.D., Korneluk R., Tremblay M.J., Angel J.B., Kumar A. (2021). Role of RIPK1 in SMAC mimetics-induced apoptosis in primary human HIV-infected macrophages. Sci. Rep..

[222] Okoye A.A., Fromentin R., Takata H., Brehm J.H., Fukazawa Y., Randall B., Pardons M., Tai V., Tang J., Smedley J. (2022). The ingenol-based protein kinase C agonist GSK445A is a potent inducer of HIV and SIV RNA transcription. PLoS Pathog..

[223] Wallet C., De Rovere M., Van Assche J., Daouad F., De Wit S., Gautier V., Mallon P.W.G., Marcello A., Van Lint C., Rohr O. (2019). Microglial Cells: The Main HIV-1 Reservoir in the Brain. Front. Cell. Infect. Microbiol..

[224] Macedo A.B., Novis C.L., Bosque A. (2019). Targeting Cellular and Tissue HIV Reservoirs With Toll-Like Receptor Agonists. Front. Immunol..

[225] Schlaepfer E., Audige A., Joller H., Speck R.F. (2006). TLR7/8 triggering exerts opposing effects in acute versus latent HIV infection. J. Immunol..

[226] Scheller C., Ullrich A., McPherson K., Hefele B., Knoferle J., Lamla S., Olbrich A.R., Stocker H., Arasteh K., ter Meulen V. (2004). CpG oligodeoxynucleotides activate HIV replication in latently infected human T cells. J. Biol. Chem..

[227] Scheller C., Ullrich A., Lamla S., Dittmer U., Rethwilm A., Koutsilieri E. (2006). Dual activity of phosphorothioate CpG oligodeoxynucleotides on HIV: Reactivation of latent provirus and inhibition of productive infection in human T cells. Ann. N. Y. Acad. Sci..

[228] Bhat K.H., Chaitanya C.K., Parveen N., Varman R., Ghosh S., Mukhopadhyay S. (2012). Proline-proline-glutamic acid (PPE) protein Rv1168c of Mycobacterium tuberculosis augments transcription from HIV-1 long terminal repeat promoter. J. Biol. Chem..

[229] Bhargavan B., Woollard S.M., Kanmogne G.D. (2016). Toll-like receptor-3 mediates HIV-1 transactivation via NFkappaB and JNK pathways and histone acetylation, but prolonged activation suppresses Tat and HIV-1 replication. Cell. Signal..

[230] Lim S.Y., Osuna C.E., Hraber P.T., Hesselgesser J., Gerold J.M., Barnes T.L., Sanisetty S., Seaman M.S., Lewis M.G., Geleziunas R. (2018). TLR7 agonists induce transient viremia and reduce the viral reservoir in SIV-infected rhesus macaques on antiretroviral therapy. Sci. Transl. Med..

[231] Borducchi E.N., Liu J., Nkolola J.P., Cadena A.M., Yu W.H., Fischinger S., Broge T., Abbink P., Mercado N.B., Chandrashekar A. (2018). Antibody and TLR7 agonist delay viral rebound in SHIV-infected monkeys. Nature.

[232] Borducchi E.N., Cabral C., Stephenson K.E., Liu J., Abbink P., Ng’ang’a D., Nkolola J.P., Brinkman A.L., Peter L., Lee B.C. (2016). Ad26/MVA therapeutic vaccination with TLR7 stimulation in SIV-infected rhesus monkeys. Nature.

[233] Del Prete G.Q., Alvord W.G., Li Y., Deleage C., Nag M., Oswald K., Thomas J.A., Pyle C., Bosche W.J., Coalter V. (2019). TLR7 agonist administration to SIV-infected macaques receiving early initiated cART does not induce plasma viremia. JCI Insight.

[234] Saxena M., Sabado R.L., La Mar M., Mohri H., Salazar A.M., Dong H., Correa Da Rosa J., Markowitz M., Bhardwaj N., Miller E. (2019). Poly-ICLC, a TLR3 Agonist, Induces Transient Innate Immune Responses in Patients With Treated HIV-Infection: A Randomized Double-Blinded Placebo Controlled Trial. Front. Immunol..

[235] Cheng L., Wang Q., Li G., Banga R., Ma J., Yu H., Yasui F., Zhang Z., Pantaleo G., Perreau M. (2018). TLR3 agonist and CD40-targeting vaccination induces immune responses and reduces HIV-1 reservoirs. J. Clin. Investig..

[236] Winckelmann A.A., Munk-Petersen L.V., Rasmussen T.A., Melchjorsen J., Hjelholt T.J., Montefiori D., Ostergaard L., Sogaard O.S., Tolstrup M. (2013). Administration of a Toll-like receptor 9 agonist decreases the proviral reservoir in virologically suppressed HIV-infected patients. PLoS ONE.

[237] Vibholm L.K., Konrad C.V., Schleimann M.H., Frattari G., Winckelmann A., Klastrup V., Jensen N.M., Jensen S.S., Schmidt M., Wittig B. (2019). Effects of 24-week Toll-like receptor 9 agonist treatment in HIV type 1+ individuals. AIDS.

[238] Vibholm L., Schleimann M.H., Hojen J.F., Benfield T., Offersen R., Rasmussen K., Olesen R., Dige A., Agnholt J., Grau J. (2017). Short-Course Toll-Like Receptor 9 Agonist Treatment Impacts Innate Immunity and Plasma Viremia in Individuals With Human Immunodeficiency Virus Infection. Clin. Infect. Dis..

[239] Clayton K.L., Collins D.R., Lengieza J., Ghebremichael M., Dotiwala F., Lieberman J., Walker B.D. (2018). Resistance of HIV-infected macrophages to CD8(+) T lymphocyte-mediated killing drives activation of the immune system. Nat. Immunol..

[240] Rainho J.N., Martins M.A., Cunyat F., Watkins I.T., Watkins D.I., Stevenson M. (2015). Nef Is Dispensable for Resistance of Simian Immunodeficiency Virus-Infected Macrophages to CD8+ T Cell Killing. J. Virol..

[241] Vojnov L., Martins M.A., Bean A.T., Veloso de Santana M.G., Sacha J.B., Wilson N.A., Bonaldo M.C., Galler R., Stevenson M., Watkins D.I. (2012). The majority of freshly sorted simian immunodeficiency virus (SIV)-specific CD8(+) T cells cannot suppress viral replication in SIV-infected macrophages. J. Virol..

[242] Clayton K.L., Mylvaganam G., Villasmil-Ocando A., Stuart H., Maus M.V., Rashidian M., Ploegh H.L., Walker B.D. (2021). HIV-infected macrophages resist efficient NK cell-mediated killing while preserving inflammatory cytokine responses. Cell Host Microbe.

[243] Clohosey M.L., Mann B.T., Ryan P.L., Apanasovich T.V., Maggirwar S.B., Pennington D.J., Soriano-Sarabia N. (2020). Comparable Vδ2 Cell Functional Characteristics in Virally Suppressed People Living with HIV and Uninfected Individuals. Cells.

[244] Garrido C., Clohosey M.L., Whitworth C.P., Hudgens M., Margolis D.M., Soriano-Sarabia N. (2018). γδ T cells: An immunotherapeutic approach for HIV cure strategies. JCI Insight.

[245] Mann B.T., Sambrano E., Maggirwar S.B., Soriano-Sarabia N. (2020). Boosting the Immune System for HIV Cure: A γδ T Cell Perspective. Front. Cell. Infect. Microbiol..

